# Treatment of Inflammatory Bowel Disease by Using Curcumin-Containing Self-Microemulsifying Delivery System: Macroscopic and Microscopic Analysis

**DOI:** 10.3390/pharmaceutics16111406

**Published:** 2024-10-31

**Authors:** Nabeela Ameer, Muhammad Hanif, Ghulam Abbas, Muhammad Azeem, Khalid Mahmood, Dure Shahwar, Ahmed Khames, Essam Mohamed Eissa, Baher Daihom

**Affiliations:** 1 Department of Pharmaceutics, Faculty of Pharmacy, Bahauddin Zakariya University Multan, Multan 60800, Pakistan; nabeela.ameer@multanust.edu.pk (N.A.); azeem@hamdard.edu.pk (M.A.); dureshahwar77777@gmail.com (D.S.); 2 Department of Pharmacy, Multan University of Science and Technology, Multan 60800, Pakistan; 3 Department of Pharmaceutics, Faculty of Pharmaceutical Sciences, Government College University Faisalabad, Faisalabad 38000, Pakistan; ghulamabbas@gcuf.edu.pk; 4 Institute of Chemical Sciences, Bahauddin Zakariya University, Multan 60800, Pakistan; 5 Department of Pharmaceutics and Industrial Pharmacy, College of Pharmacy, Taif University, Taif 21944, Saudi Arabia; a.khamies@tu.edu.sa; 6 Department of Pharmaceutics and Industrial Pharmacy, Faculty of Pharmacy, Beni-Suef University, Beni-Suef 62514, Egypt; essam.mohamed@pharm.bsu.edu.eg; 7 Department of Pharmaceutics and Industrial Pharmacy, Cairo University, Cairo 11562, Egypt; baher.daihom@pharma.cu.edu.eg; 8 Pharmaceutical Engineering and 3D Printing (PharmE3D) Lab, Division of Molecular Pharmaceutics and Drug Delivery, College of Pharmacy, University of Texas at Austin, Austin, TX78712, USA; 9 College of Pharmacy, American University of Iraq-Baghdad, Baghdad 10071, Iraq

**Keywords:** curcumin, self-microemulsifying drug delivery system, colon, acute toxicity, inflammatory bowel disease

## Abstract

**Background:** The lack of local availability for drugs in the colon can be addressed by preparing a self-microemulsifying drug delivery system (SMEDDS) of curcumin (Cur) which is ultimately used for the treatment of inflammatory bowel disease (IBD). **Methods:** From preformulation studies, Lauroglycol FCC (oil), Tween 80 (surfactant), Transcutol HP (co-surfactant), and Avicel (solid carrier) were selected for the preparation of blank liquid and solid Cur-loaded SMEDDSs (S-Cur-SMEDDSs). **Results:** Z-average size (12.36 ± 0.04 nm), zeta potential (−14.7 ± 0.08 mV), and polydispersity index (PDI) (0.155 ± 0.036) showed a comparative droplet surface area and charge of both SMEDDSs. The physicochemical stability of Cur in S-Cur-SMEDDSs was confirmed via FTIR, DSC, TGA, and XRD analyses, while morphological analysis through SEM and atomic force microscopy (AFM) confirmed Cur loading into SMEDDSs with an increased surface roughness root mean square (RMS) of 11.433 ± 0.91 nm, greater than the blank SMEDDS. Acute toxicity studies with an organ weight ratio and % hemolysis of 15.65 ± 1.32% at a high concentration of 600 mM showed that S-Cur-SMEDDSs are safe at a medium dose (0.2–0.8 g/kg/day). The excellent in vitro antioxidant (68.54 ± 1.42%) and anti-inflammatory properties (56.47 ± 1.17%) of S-Cur-SMEDDS proved its therapeutic efficacy for IBD. Finally, S-Cur-SMEDDS significantly improved acetic acid-induced IBD in albino rats through a reduction in the disease activity index (DAI) and macroscopic ulcer score (MUS) from 4.15 ± 0.21 to 1.62 ± 0.12 at 15 mg/kg/day dose, as confirmed via histopathological assay. **Conclusions:** Based on the above findings, S-Cur-SMEDDS appears to be a stable, less toxic, and more efficacious alternative for Cur delivery with strong competence in treating IBD.

## 1. Introduction

Inflammatory bowel disease (IBD) is a chronic inflammatory disorder involving a disrupted intestinal barrier, inflamed colonic areas, and high mucus production, leading to changes in mucus-secreting cells, ultimately resulting in an ulcer [1]. The dysregulation of mucosal immunity in IBD patients leads to an excessive production of inflammatory cytokines and leukocytes into the bowel, resulting in uncontrolled intestinal inflammation. For the treatment of IBD, anti-inflammatory drugs such as certain amino salicylates, corticosteroids, immunosuppressive agents, and biological compounds (monoclonal immunoglobulins) are commonly used. However, due to their systemic administration, these drugs cause many side effects (diarrhea, lymphopenia, etc.). So, the targeted therapy of inflamed colonic areas could be an effective approach to treating IBD along with reducing systemic adverse effects and improving therapeutic effectiveness [2].

Nano-sized oral drug delivery systems are preferred in treating IBD due to their targeted deposition in inflamed areas of the intestine [3]. Among the nanocarriers described thus far, lipid-based drug delivery systems have been frequently investigated for improving the oral bioavailability of hydrophobic drugs such as curcumin (Cur). Therefore, recent approaches have noted that combining lipid-based excipients with anti-inflammatory drugs within a nanocarrier, such as a self-microemulsifying drug delivery system (SMEDDS), could be a useful technique because patients with this disease lack physiological lipids, and several lipids have immunomodulatory properties [4].

Although synthetic drugs currently dominate the market, the aspect of toxicity caused by these drugs cannot be ruled out. Their long-term use has many adverse effects, and nowadays, treatment with natural drugs like Cur for their anti-inflammatory effects have gained much interest, leading to the discovery of dosage forms containing Cur which, in addition to the property of retaining protein denaturation, have many other benefits with minimal side effects [5].

Cur has a wide variety of therapeutic effects against many inflammatory disorders along with hepatoprotective and antioxidant properties; despite its numerous pharmacological benefits, its applications are severely limited due to low aqueous solubility, instability in the gastrointestinal tract, rapid metabolism, and relatively low bioavailability. Thus, increasing Cur solubility has the potential to increase its bioavailability and aid in the reduction in the dose required to inhibit inflammatory disorders. The FAO and WHO Expert Committee on Food Additives reported in 1996 that the acceptable daily intake of Cur is up to 3 mg/kg body weight. Cur as a natural product has a long history of consumption in the human diet, but there appear to be few scientific studies on its toxicity to both animals and humans, especially at high doses [6].

The objective of the present study was to synthesize solid Cur SMEDDS (S-Cur-SMEDDS) for improving acetic acid-induced IBD in albino rats. A prepared S-Cur-SMEDDS was initially evaluated for % reconstitution efficiency, Z-average droplet size, zeta potential, polydispersity index (PDI), and in vitro % release using sink conditions. This study conducted further explorations by measuring in vitro free radical scavenging activity and anti-inflammatory activity using bovine serum albumin and ovalbumin to demonstrate the beneficial antioxidant effect of formulations. In order to investigate the safety profile of the developed formulation, an acute toxicity study using the organ weight ratio and % hemolysis on albino mice was performed. To analyze the efficacy of a solid Cur SMEDDS for the treatment of acetic acid-induced IBD in albino rats, a macroscopic evaluation of colon damage was conducted, aided by microscopic/histopathological analysis before and after treatment, to provide insight into the utilization of a Cur-loaded solid SMEDDS to treat IBD.

## 2. Materials and Method

Curcumin (C_21_H_20_O_6_, M.W. ≈ 368.38 g/mol) was obtained from Acros-Organics, Newark, NJ, USA, and Lauroglycol FCC, Shanghai, China (Propylene glycol monolaurate, HLB ≈ 5) and Transcutol HP (Diethylene glycol mono-ethyl ether, HLB ≈ 4) were gifted from Gattefosse SAS, Saint-Priest, France. Polyethylene glycol (PEG 400) M.W. ≈ 62.07 g/moL, Tween 80 (Polyoxyethylene sorbitan monooleate, M.W. ≈ 1310 g/moL, HLB ≈ 15), Avicel (M.W. ≈ 370.35 g/moL), potassium dihydrogen phosphate (M.W. ≈ 136.086 g/moL), sodium hydroxide (NaOH) M.W. ≈ 39.997 g/moL, hydrogen peroxide (H_2_O_2_), acetic acid (CH_3_COOH) MW ≈ 60.052 g/moL, and diethyl ether (C_2_H_5_)_2_O M.W. ≈ 74.12 g/moL, were obtained from Sigma Aldrich, Merck, Darmstadt, Germany, along with 2,2-diphenylpicrylhydrazine (DPPH) (Alfa Aesar by Thermo Fisher Scientific, Bridgewater, NJ, USA) and bovine serum albumin (BSA) M.W. ≈ 66430.3 g/moL (BioWorld, BioPLUS Fine Research Chemicals, Dublin, OH, USA). Hydrochloric acid (MW ≈ 36.46 g/moL) was obtained from AnalaR^®^, BDH Chemicals, Poole, UK. Dimethyl sulfoxide DMSO was acquired from DUKSAN Pure Chemicals, Ansan, Republic of Korea. Diclofenac sodium (M.W. ≈ 318.13 g/moL) and Mesalamine (5-amino salicylic acid derivative) were gifted by Getz Pharma, Multan, Pakistan. Double-distilled water was used during the complete study. All above-mentioned chemicals, as well as reagents, were of analytical standard.

### 2.1. Preparation of Cur SMEDDS

Cur-loaded liquid SMEDDSs (L-Cur-SMEDDS) were prepared via the previously reported work of Hanif et al. [7]. Briefly, Lauroglycol FCC (oil), Tween 80 (surfactant), and Transcutol HP (co-surfactant) were mixed in a ratio of 1:6:3, respectively, in a Thermomixer (Eppendorf, Hamburg, Germany) at 100 rpm for 10 min at 70 °C, and Cur (5% *w*/*w*) was then loaded with continuous stirring until homogenous. Adsorption onto the surface of the solid carrier technique was employed for the preparation of Cur-loaded solid SMEDDSs via a slight modification of the already-reported method of Kamal M.M. et al. [8]. Briefly, L-Cur-SMEDDS was added dropwise in a pestle and mortar containing the inert solid adsorption carrier Avicel in a fixed ratio (1:2) at room temperature (25 ± 0.75 °C) and mixed well until free flowing powder of S-Cur-SMEDDS was obtained.

### 2.2. Reconstitution Efficiency, Z-Average Droplet Size and Physical Stability

The reconstitution efficiency (%RE) of the prepared SMEDDS was investigated via the method described by Matsaridou I. et al. [9]. Briefly, 500 mg of S-Cur-SMEDDS was reconstituted in 100 mL phosphate buffer solution (PBS) pH 7.4 kept under magnetic stirring (100 rpm) at 37 ± 0.5 °C for 3 h. The 5 mL aliquot samples were collected at predetermined time intervals (5 and 30 min), centrifuged for 5 min at 2000 rpm, and filtered through 0.45 µm filter membrane, and the filtrate was then tested for % transmittance (%T) at 425 nm using a UV-vis spectrophotometer (Bruker, Alpha UV 1900, Karlsruhe, Germany). To exclude any interference caused by other components of the medium, Cur solution was prepared in PBS (pH 7.4) as standard, %T values were measured [10], and %RE was calculated by following Equation (1):

(1) $$\%RE=\frac{\left({\%T}_{ss}-{\%T}_{B}\right)}{{(\%T}_{SL}-{\%T}_{B})}\times 100,$$

where T_ss_, T_B_, and T_SL_ are % transmittance of the reconstituted S-Cur-SMEDDS, Cur solution, and L-Cur-SMEDDS, respectively. The experiments were performed in triplicate (mean ± SD).

Blank liquid SMEDDS and S-Cur-SMEDDS were diluted separately 1000 fold with deionized water and mixed prior to measurement. The Z-average droplet size, zeta potential, and polydispersity index (PDI) of the resulting emulsions after reconstitution were measured using a Zetasizer Nano ZS analyzer (Model ZEN3600, Malvern Instruments, Malvern, UK). All the experiments were performed in triplicate (mean ± SD).

### 2.3. Physicochemical Characterization

FTIR spectroscopic analysis was carried out to investigate possible chemical interactions between Cur and SMEDDS, which were observed as a shift or broadening of the peaks of characteristic chemical groups. The FTIR spectra of excipients (Lauroglycol FCC, Tween 80, Transcutol HP, and solid carrier Avicel) Cur, and S-Cur-SMEDDS were determined via ATR-FTIR (Bruker, United States America) with absorbance spectra recorded in the range of 4000 to 400 cm^−1^ [11]. Differential scanning calorimetry (DSC) and thermogravimetric analysis (TGA) were used to evaluate the thermochemical properties of S-Cur-SMEDDS with the optimum temperature ramp speed adjusted to 10 °C/min, expelled at flow rate 70 mL/min, with pure dry nitrogen and heat flow from 30 to 230 °C. X-ray powder diffraction (XRD) analysis was also performed to analyze the physical state of Cur, Avicel, and S-Cur-SMEDDS in a high-resolution powder diffractometer (Q600 V20.9, TA Instruments, New Castle, DE, USA) equipped with a scintillation counter detector and divergent beam [12].

Scanning electron microscopic (SEM) analysis of Cur, liquid SMEDDS before solidification, and S-Cur-SMEDDS at different magnifications was performed using a scanning electron microscope (Jeol, Tokyo, Japan). Additionally, for surface structure and morphological assessment of SMEDDS, an atomic force microscope (AFM, Digital Instruments, Tonawanda, NY, USA) mounted with a J scanner and silicon sensor (TESP, spring constant of 2080 N/m, Bruker) was used to capture images with a resolution of 256256 pixels and a scan rate of 0.51.0 Hz. The mean particle heights, width, and surface roughness of S-Cur-SMEDDS were measured [13].

### 2.4. In Vitro % Release

In vitro dissolution studies of Cur and S-Cur-SMEDDS were performed by a method already described by Hanif et al. [7]. Briefly, Cur (100 mg) was placed in dissolution apparatus containing 900 mL PBS (pH 7.4) and PEG 400 (10% *v*/*v*), maintained at 37 ± 0.1 °C with a stirring speed of 75 rpm. PEG 400 was added to provide sink conditions that prevented accumulation of Cur in the dissolution medium released from SMEDDS as previously reported by Phillips D. J. et al. [14]. Samples were collected periodically and replaced with fresh PBS (pH 7.4). The 5 mL aliquot samples were withdrawn after specific time intervals and filtered through Whatman filter paper. The filtrate was diluted to 5 mL with PEG 400, and absorbance was measured via a UV spectrophotometer (Bruker Germany UV 1900) at 425 nm wavelength. Cur concentration in samples was determined via the already-developed standard curve of Cur. A similar procedure was repeated for dissolution studies of S-Cur-SMEDDS (*n* = 3).

### 2.5. Free Radical Scavenging Activity

For the DPPH radical scavenging activity of Cur, the method of Afolayan et al. [15] was adopted with some modifications. Briefly, 2 mL 0.135 mM ethanolic solution of DPPH was mixed with each already-prepared concentration of S-Cur-SMEDDS (equivalent to 0.002–20 µg/mL Cur). The resulting mixtures were vortexed for 5 min and placed in the dark for 30 min at room temperature, and absorbances were measured spectrophotometrically at 517 nm at different time intervals (mean ± SD) (*n* = 3). For antioxidant analysis via hydrogen peroxide, a similar procedure was repeated except mixing with 1 mL of 4 mM H_2_O_2_ solution in PBS (pH 7.4) instead of using DPPH (mean ± SD) (*n* = 3). Both radical scavenging activities by DPPH and H_2_O_2_ were calculated by following Equation (2):

(2) $$\%\;scavenging\;activity=\left[\frac{{AD}_{C}-{AD}_{S}}{{AD}_{C}}\right]\times 100,$$

where AD_C_ is the absorbance of the DPPH solution as control, and AD_S_ is the absorbance of S-Cur-SMEDDS in ethanol at different concentrations [16].

### 2.6. In Vitro Anti-Inflammatory Effect

In vitro anti-inflammatory activity was determined via the protein denaturation method using bovine serum albumin (BSA). Briefly, 1% *w*/*v* aqueous solution of BSA was mixed separately with already-prepared solutions of S-Cur-SMEDDS in water and Cur in ethanol (0.25 ppm each), and the pH of the resultant mixtures was adjusted to 6.3 with dilute HCl. The turbidity of the resulting solutions was observed using a UV spectrophotometer at 660 nm, and denaturation of BSA was calculated via the already-prepared BSA solution in saline buffer (0.9% *m*/*v*), considered as control. The same concentration of diclofenac sodium solution (0.05 mL each) was used as standard, and % inhibition was calculated via Equation (3). The experiment was repeated in triplicate (*n* = 3) for mean ± SD [17].

(3) $$\%\;inhibition=\left(\frac{Vt}{Vc}-1\right)\times 100,$$

where Vt is the absorbance of test samples (S-Cur-SMEDDS and Cur), and Vc is the absorbance of control (BSA).

### 2.7. Acute Toxicity Studies

Acute oral toxicity studies of Cur and S-Cur-SMEDDS were performed via a slightly modified version of the method of Jantawong et al. [18]. Briefly, 24 male albino mice (body weight 25–50 g) were randomly divided into five groups (Group I and Group III (3 mice each) and Group II, IV, and V (6 mice each)). After approval from the Animal Ethical Committee of Bahauddin Zakariya University Multan, Pakistan (04/UREC/2021), all animals were kept in hygienic laboratory conditions at 23 ± 3 °C, relative humidity 30–60%, and 12 h light/dark cycles with access to food and water. Group I was kept as a control with a normal diet, Group II received a single oral dose of Blank S-SMEDDS (3 mice 8 g/kg and 3 mice 15 g/kg), Group III was given a single oral low dose of S-Cur-SMEDDS (0.05 g/kg equivalent to 1.25 mg Cur), and Group IV was given medium oral doses of 0.2 g/kg to 3 mice and 0.4 g/kg to the other 3 mice, equivalent to 5 mg/kg and 10 mg/kg Cur, respectively. Finally, group 5 was given single high oral doses of S-Cur-SMEDDS (2 g/kg to 3 mice and 4 g/kg to the other 3 mice, equivalent to 50 mg/kg and 100 mg/kg Cur, respectively). All doses of SMEDDS were given to mice via an oral gavage tube diluted with distilled water within 10 min. For acute toxicity studies, clinical signs of tremors, excitability, salivation depression, morbidity, and mortality were observed twice daily for 14 days after treatment [19].

#### Ex Vivo Hemolytic Assay

Blood compatibility with respect to Cur and S-Cur-SMEDDS on human blood was analyzed via the already-reported method of Azeem et al. [20], with a slight modification. Briefly, solutions of different concentrations (50, 100, 200, 400, and 600 μM) of Cur and S-Cur-SMEDDS in PBS (pH 7.4) were prepared separately, where for Cur, 1% DMSO was added, and all solutions (5 mL each) were mixed with 300 μL of anti-coagulated fresh human blood and centrifuged at 4500 rpm for 5 min after incubation at room temperature for 90 min. The absorbance of the supernatant was analyzed via a UV spectrophotometer (Bruker Germany UV 1900) at 545 nm. A total of 0.1% Na_2_CO_3_ was used as a negative control for 100% hemolysis, and 0.1% Triton X-100 was used as a positive control. All experiments were repeated in triplicate for mean ± SD (*n* = 3). %Hemolysis was calculated via using the following equation:

(4) $$\%\;hemolysis=\frac{A_{S}-A_{N}}{A_{N}}\times 100,$$

where A_S_ and A_N_ denote the absorbance of the samples (Cur and S-Cur-SMEDDS) and negative control, respectively.

### 2.8. In Vivo Assessment of S-Cur SMEDDS for IBD Treatment

Twenty-four male albino rats, aged 8 to 15 weeks and weighing between 150 and 250 g, were split into 4 groups (I, II, III, IV) (*n* = 6). The study protocol was approved by the Research Ethics Committee of the Faculty of Pharmacy, Bahauddin Zakariya University, Multan. ICH guidelines were followed to conduct animal studies. All animals were housed at 25 ± 0.50 °C, relative humidity 40–60%, with alternate light and dark cycles of 12 h, free access to water ad labium, and a standard diet. Group I was kept as control with no treatment. Rats in Groups II, III, and IV were anaesthetized lightly with diethyl ether after overnight fasting, and IBD was induced via intra-rectal administration of 1 mL acetic acid (4% *v*/*v*) at 6 cm proximal to the anus for 30 s. To flush the colon, 1 mL PBS (pH 7.4) was also administered similarly. Group II was kept as the negative control after the induction of IBD. Group III and IV were administered mesalamine (2 mg/60kg) and S-Cur-SMEDDS (15 mg/Kg/day) orally, respectively, for 3 days.

#### 2.8.1. Macroscopic Evaluation of Colonic Damage

Rats of all Groups (I–IV) were weighed daily and observed for weight loss, stool consistency, and anal bleeding. %Weight loss on each day in relation to initial weight was calculated via Equation (5):

(5) $$\%\;WL=\left[\frac{W_{D}-W_{i}}{W_{i}}\right]\times 100,$$

where % WL is the percent weight loss of the rat, W_D_ is the weight change of each rat on day 0, 2, 4, and 7, and W_i_ is the initial weight before the experiment [21].

Disease activity index (DAI) or macroscopic damage on each day was calculated, with a slight modification, as a combined score of weight loss, stool consistency, and bleeding via Equation (6):

(6) $$DAI=\frac{WL+SC+BL}{3},$$

where WL is the average weight loss score, SC is the stool consistency score, and BL is the bleeding score calculated from Table 1.

For macroscopic evaluation of colonic inflammation, immediately after the 7th day, rats were anesthetized by being kept in euthanizing box and euthanized via cervical dislocation, and colons were excised 6 cm above the anal margin. Contents of colonic gut lumen were removed by flushing with PBS (pH 7.4) using a blunt needle attached to a syringe, and inflammation was observed via macroscopic analysis. Each colon length to the nearest mm was measured, cut longitudinally, rinsed with PBS (pH 7.4), and dried on filter paper, and after weighing (g), each animal’s colon weight-to-length ratio was measured [22].

#### 2.8.2. Histopathological Examination

All colon samples (Group I–IV) after macroscopic observations were excised subsequently for microscopic examination based on the scoring system. Each sample was fixed in 10% formalin in PBS (pH 7.4) for 1 week, after which it was washed with distilled water for 2 h. Histopathological assessment of the extent of inflammation in the colon was assessed via cut sections mounted onto slides stained with H&E using the standard technique and were evaluated via the microscopic scoring criteria (MiSC), where 0 = normal; 1 = slight increase in cellularity (lymphocytes in lamina propria); 2 = increase in cellularity, neutrophils present, and mild edema; 3 = diffuse increase in cellularity, focal erosions, or ulcerations in mucosa; 4 = increased cellularity and large or multifocal ulcerations; and 5 = diffuse ulcerations and loss of mucosal structure. Additionally, histopathological slides were also assessed on the basis of anatomic changes in the colonic tissue membrane of all rats groups [23].

### 2.9. Statistical Analysis

All data were represented as mean ± SD (*n* = 6), and changes in the parameters among the treated groups were analyzed via the one-way ANOVA (*p* ≤ 0.05).

## 3. Results and Discussion

### 3.1. Self-Emulsification and Physical Stability Analysis

%RE of prepared S-Cur-SMEDDS in PBS (pH 7.4) based on transmittance (%T) was 86 ± 1.42, which gradually increased to 97 ± 1.15% with the passage of time, suggesting solvent penetration into SMEDDS. In addition, intermolecular interactions between excipients (Lauroglycol FCC, Tween 80, Transcutol HP, and solid carrier Avicel) can significantly affect the reconstitution efficiency of SMEDDS, playing an important role in the maintenance of emulsification properties. The z-average globule size of blank SMEDDS and S-Cur-SMEDDS after reconstitution was 10.66 ± 0.16 and 12.36 ± 0.04 nm (Figure S1), respectively, while zeta potential upon dilution was −11.4 ± 0.216 and −14.7 ± 0.08 mV, respectively. The results showed that Z-average droplet size aligns with the characteristics of the self-microemulsifying drug delivery system (SMEDDS). Microemulsions so formed have droplet sizes less than 100 nm and belong to Type IIIa according to the Pouton classification of lipid formulations. More negative zeta potential correlated with comparatively larger droplet sizes confirmed the loading of Cur into SMEDDS. The robustness of Cur-SMEDDS through PDI was tested by diluting it several times, even after 1000 folds showed no phase separation and precipitation and no significant change in PDI (0.155 ± 0.036), indicating the physical stability of the S-Cur-SMEDDS [24].

### 3.2. Chemical Stability and Interaction Studies

**Figure 1:**
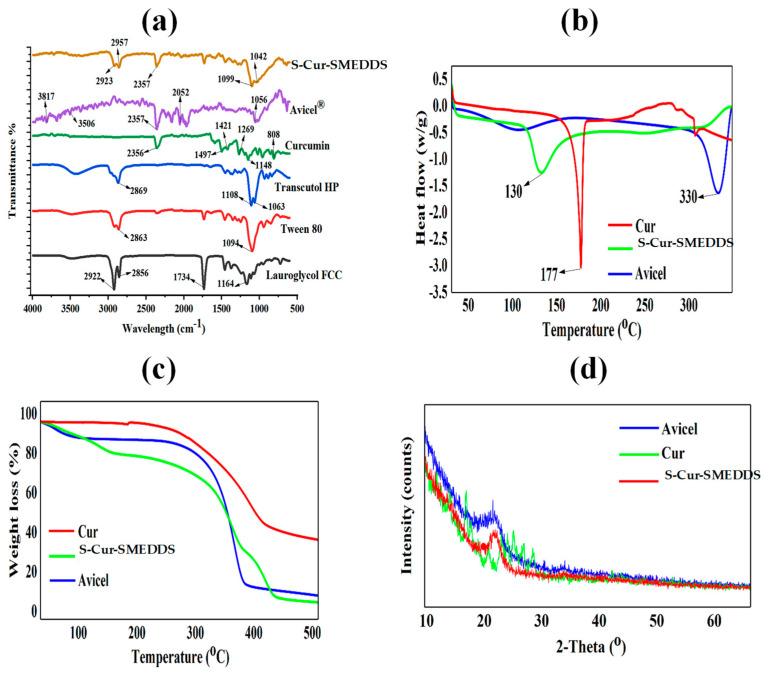
(**a**) ATR-FTIR spectra of Lauroglycol FCC, Tween 80, Transcutol HP, Avicel, Cur, and S-Cur-SMEDDS showed no chemical interactions. (**b**) DSC thermograms and (**c**) TGA analysis confirmed the absence of crystalline peaks of Cur after loading into SMEDDS. (**d**) X ray diffraction studies indicated no traces of crystalline peaks of Cur in S-Cur-SMEDDS.

In FTIR analysis, a shift in the vibrational bands from the original location, the appearance or loss, and the broadening or sharpening of peaks is an indication of the chemical interaction of Cur with excipients. From Figure 1a, FTIR spectra of Lauroglycol FCC, Tween 80, Transcutol HP, Avicel, Cur, and S-Cur-SMEDDS indicated prominent bands of Cur at (1) 1600 cm^−1^ for stretching vibration of the benzene ring skeleton; (2) 1510 cm^−1^ to mixed (C=O) and (C=C) vibration; (3) 1425 cm^−1^ to olefinic C–H in-plane bending vibration (δC–H); and (4) 1280 cm^−1^ to Ar–O stretching vibration. The broad absorption bands at 3410 cm^−1^ arise from the stretching mode of OH groups. Bands in the range of 3079–3000 cm^−1^ can be attributed to the aromatic C–H stretching vibration. Similar spades were observed in S-Cur-SMEDDS, showing no change in peaks of Cur even after being loaded into SMEDDS [25]. Furthermore, in the FTIR spectra of S-Cur-SMEDDS, all the significant stripes of excipients persisted, and there was no interference, showing no chemical interaction of Cur with excipients. Avicel showed characteristic bands at 2334 cm^−1^ corresponding to -CH_2_- stretching and at 2865 and 1146 cm^−1^. In S-Cur-SMEDDS, the broadening and shifting of peaks at 1216 cm^−1^ was attributed to no physical interaction and was considered relatively stable [26].

### 3.3. Thermal Stability for Melting Temperature Analysis

DSC is a commonly used thermoanalytical technique for studying melting temperatures, material transitions from crystalline to amorphous states, material decomposition, and drug excipient compatibility. From the DSC thermogram of Cur, Avicel, and S-Cur-SMEDDS, it was observed that Cur exhibited a sharp melting endothermic peak at 177.8 °C, corresponding to its melting point (Figure 1b). On the other hand, the absence of a sharp endotherm in the DSC thermogram of Avicel and S-Cur-SMEDDS confirmed its porous amorphous nature. No peaks corresponding to Cur crystals were observed, indicating the absence of the crystalline nature of Cur after its loading into SMEDDS, thus confirming that the preparation method did not have a negative impact on the dissolution of Cur embedded in SMEDDS or induce crystal growth and precipitation. Solubilization of Cur crystals within blank SMEDDS during heating may result in an obtuse peak with reduced intensity, suggested that when Cur is loaded into the SMEDDS, its physical nature changes from crystalline to amorphous/molecular dispersion due to the solubilization of Cur, which further improves its bioavailability. The absence of a crystalline peak is also due to the fact that Cur has likely dissolved in SMEDDS, resulting in the disappearance of its crystalline structure [27].

The TGA of Cur, Avicel, and S-Cur-SMEDDS shown in Figure 1c demonstrated negligible weight loss/gradual thermal degradation from room temperature to 150 °C. This is an indication that no solvent evaporated during sample heating above 100 °C, and SMEDDS did not contain water during preparation. Maximum decomposition was observed above 300 °C as more than 50% weight loss was observed at this temperature, confirming that Cur was stable even after loading into SMEDDS [28].

### 3.4. X-Ray Powder Diffractometry

X-ray diffractometry analysis was performed to characterize the physical state of Cur, Avicel, and S-Cur-SMEDDS. As shown in Figure 1d, typical diffraction peaks of Cur were visible between 10° to 30°, and Avicel showed peaks between 21° and 25°; however, no trace of the typical crystalline peaks of Cur was observed for solid SMEDDS (S-Cur-SMEDDS); instead, there were Avicel peaks, proving the non-crystalline appearance of Cur after its embedding into the SMEDDS [29]. Results also indicated that in solid SMEDDS, Cur remained in a molecular state and transformed into an amorphous form, which was responsible for the highest solubilization capacity and a faster rate of dissolution with increased stability [30].

### 3.5. Surface Structure and Morphological Analysis

**Figure 2:**
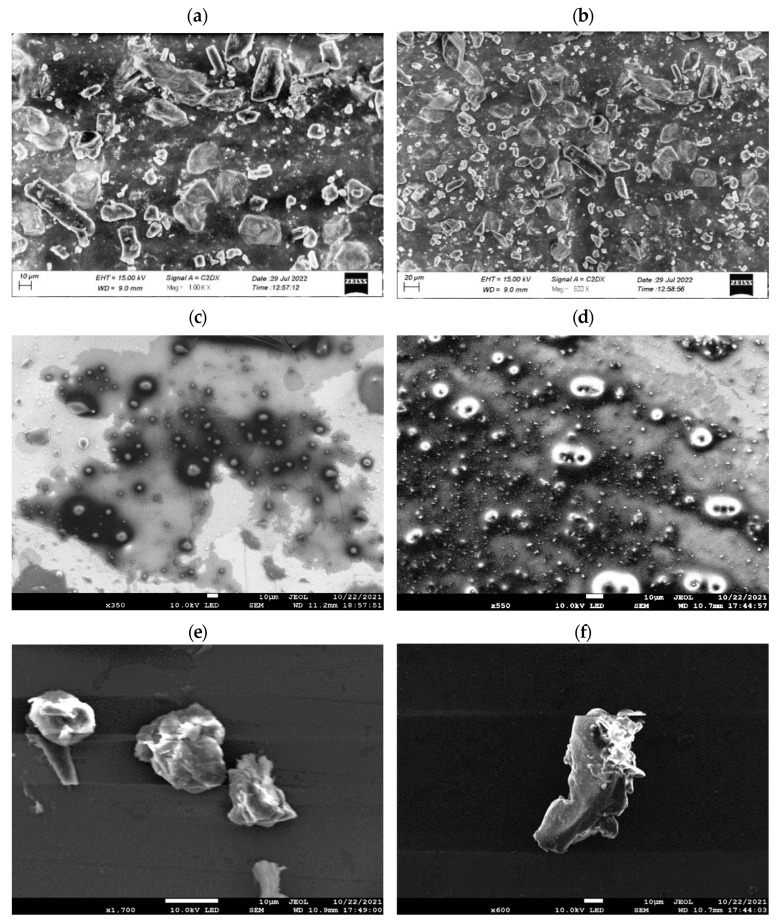
SEM images of (**a**,**b**) Cur under 100 KX and 600× in the 10–20 µm size tange. (**c**) Blank L-SMEDDS under 200× magnification in the 100 µm size range. (**d**) L-Cur-SMEDDS at 350× and 550× in the 10 µm size range showing an amorphous nature. (**e**,**f**) S-Cur-SMEDDS under 550× magnification in the 10 µm size range showing confirmation of amorphous properties.

SEM micrographs of Cur in Figure 2a,b proved that crystalline particles with very low aqueous solubility limit their therapeutic effectiveness. However, the SEM images of blank liquid SMEDDS and Cur-loaded liquid SMEDDS shown in Figure 2c,d demonstrated the embedding of Cur in SMEDDS, whereas S-Cur-SMEDDS at different magnifications illustrated the nanosized range particles with porous surfaces due to the presence of Avicel, resulting in the increased surface area of particles (Figure 2e,f). It was seen from the results that liquid Cur SMEDDS were successfully solidified using Avicel, with minimum agglomeration between solid particles [31].

**Figure 3:**
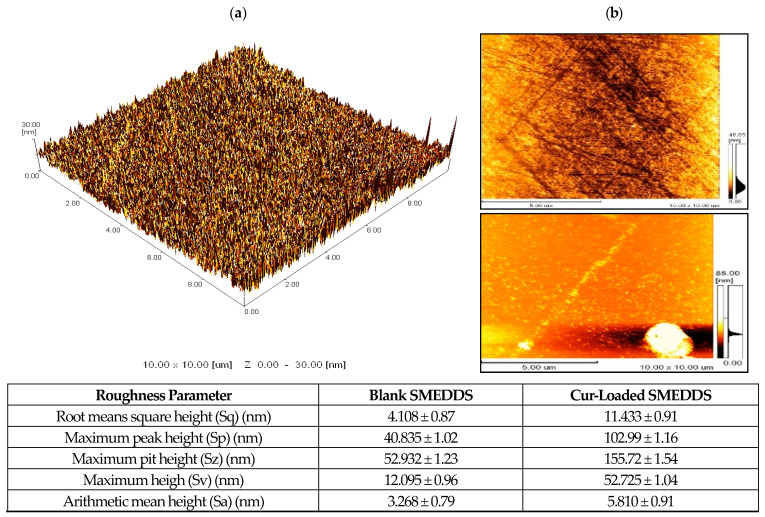
AFM analysis showing (**a**) surface roughness of Cur-loaded SMEDDS; (**b**) AFM of blank and Cur-loaded SMEDDS with calculated roughness parameters.

For investigating morphological changes through AFM, the observed roughness root mean square (RMS) of blank liquid SMEDDS was 4.108 ± 0.87 nm and smooth, whereas the cell boundary of S-Cur-SMEDDS became slightly rougher after the encapsulation of Cur with RMS, increasing to 11.433 ± 0.91 nm (Figure 3a), showing no signs of aggregation due to the adsorbed droplets of SMEDDS on the solid carrier Avicel. The maximum mean height of droplets of blank SMEDDS was 40.835 ± 1.02 nm, which was lower than Cur-loaded SMEDDS, having a 102.99 ± 1.16 nm mean height, showing the entrapment of Cur in SMEDDS. The reason behind this is the flattening of blank SMEDDS droplets on the detector surface, while due to the presence of Cur and solid carrier, Avicel increased the height of droplets [32].

The comparative two-dimensional images of the blank and Cur-loaded SMEDDS surface analysis shown in Figure 3a,b indicated a comparison corresponding to the width and height of the peaks. At calculated maximum widths of 2.49, 1.96, and 1.92 µm on the x-axis, the peak widths of the three obtained peaks of blank SMEDDS ranged between 0.06 and 0.12 µm. Similarly, at calculated maximum heights of 9.12, 9.63, and 10.38 nm on the y-axis, the resulting peak heights were in the range of 0.08–2.49 nm (Figure 3) [33]. On the other hand, in the case of the Cur-loaded SMEDDS at maximum widths of 2.05, 2.09, and 1.51 µm, the three peaks obtained were present in the range between 0.06 and 0.11 µm, and at calculated maximum heights of 11.16, 15.13, and 16.12 nm, the resulting peak heights ranged between 0.01 and 3.54 nm (Figure 3). It can be inferred from the results that the globule size of the Cur-loaded SMEDDS was more than the blank SMEDDS, with more surface roughness, indicating the successful incorporation of Cur into SMEDDS [33].

### 3.6. In Vitro % Cur Release

**Figure 4:**
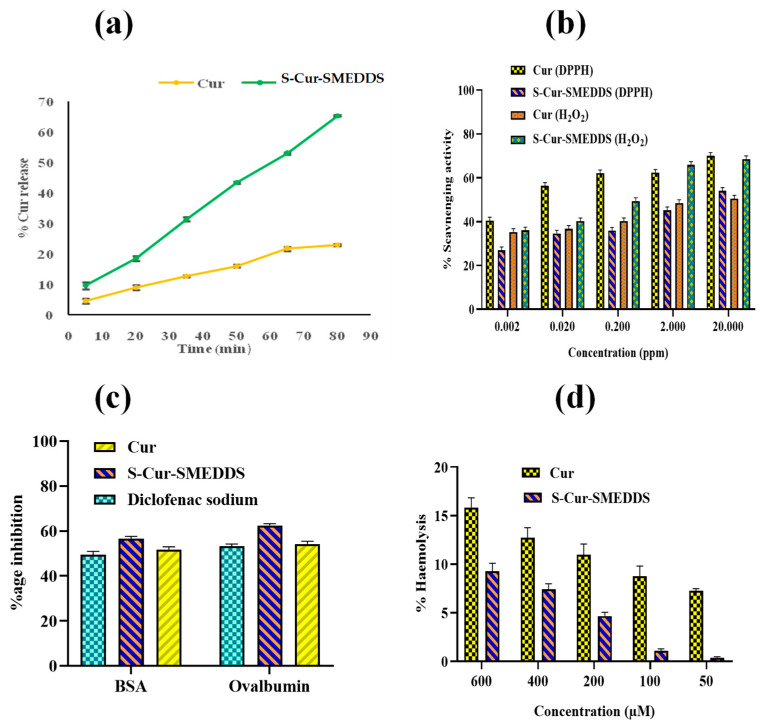
(**a**) Represented % release of Cur through S-Cur-SMEDDS in sink conditions (*n* = 3). (**b**) Concentration-dependent in vitro % scavenging activity of Cur and S-Cur-SMEDDS with DPPH and hydrogen peroxide (H_2_O_2_) (*n* = 3). (**c**) In vitro anti-inflammatory effect of Cur, S-Cur-SMEDDS, and reference standard diclofenac sodium via protein denaturation of BSA and ovalbumin (*n* = 3). (**d**) % Hemolytic effect for investigation of toxicity of Cur and S-Cur-SMEDDS against blood cells (*n* = 3).

Cur, due to its having very poor aqueous solubility, needs sink conditions such as 10% *v*/*v* PEG 400 as a receptor medium. The selection of organic polymer was based on considering the partition coefficient of the selected drug between the aqueous dissolution phase and organic polymer PEG. The dissolution profiles of Cur and SMEDDS formulation are shown in Figure 4a. It was observed from results that at the end of 80 min, Cur release 65.42 ± 1.43% from S-Cur-SMEDDS was maximum; however, % release of Cur showed 22.81 ± 1.72% at 80 min, showing faster Cur release from S-Cur-SMEDDS. The reason behind this is that SMEDDS is composed of a mixture of both Tween 80 and Transcutol HP (2:1), the former of which improved release by enhancing the rate of drug solubilization into gastrointestinal fluids, while the latter helped in dispersing lipophilic Cur molecules into fine droplets of the oil phase (Lauroglycol FCC) in SMEDDS. Thus, a combination of surfactant (Tween 80) and co-surfactant (Transcutol HP) helped in achieving a fine dispersion of Cur in oil, as well as increased aqueous dispersion and solubility, which permits a faster rate of Cur release into the aqueous phase, which, in turn, affects bioavailability [34]. From the previous literature, it was observed that upon oral administration, SMEDDS, which typically consists of curcumin dissolved in a mixture of oils, surfactants, and co-surfactants, does not release the drugs easily in the stomach. The small droplet size of SMEDDS ensures enhanced solubility and stability, which protect curcumin from degradation in the stomach and upper GIT, allowing it to be transported intact into the colon. Furthermore, emulsified droplets can also bypass enzymatic degradation and avoid premature absorption in upper GIT, ensuring that curcumin reaches the colon [7,35,36].

### 3.7. Free Radical Scavenging Activity

The free radical scavenging activity of Cur and S-Cur-SMEDDS was evaluated by measuring changes in the absorbance and color caused by DPPH reduction. The decolorization of DDPH solution depends on the number of electrons captured and the decided strength of the antioxidant properties of the compounds under investigation. DDPH, a free radical, can usually combine with hydrogen donors like Cur, resulting in decreased UV absorbance. The % scavenging activity of Cur against DPPH (Figure 4b) ranged from 40.48 ± 1.43% to 69.97 ± 1.51%, indicating that Cur scavenging activity is attributed to its high reducing power, and higher phenolic contents have the ability to convert DPPH into a non-radical form DPPH-H during reaction. Cur after incorporation into S-Cur-SMEDDS retained its antioxidant properties ranging from 26.95 ± 1.31% to 54.06 ± 1.47%, comparable to Cur with the advantage of improved solubility and dissolution of Cur SMEDDS in GI fluids [37].

Furthermore, as H_2_O_2_ is an important oxidative species that destroys cells through DNA damage, peroxidation was a dominant mode of oxidative degradation because the oxygen atom of H_2_O_2_ undergoes oxidation, resulting in free radical formation. As shown in Figure 4b, Cur and S-Cur-SMEDDS had effective H_2_O_2_ scavenging ability at a concentration of 20 µg/mL, which was found to be 50.49 ± 1.48% and 68.54 ± 1.42%, respectively. The obtained results confirmed that after loading into S-Cur-SMEDDS, Cur has an efficient scavenging activity for free radicals [38].

### 3.8. In Vitro Anti-Inflammatory Effect

The in vitro anti-inflammatory effect of Cur and S-Cur-SMEDDS was analyzed via the denaturation of BSA and ovalbumin. Protein denaturation has been linked to the occurrence of inflammatory responses, leading to a variety of inflammatory diseases such as IBD. As a result, the ability of a compound to inhibit protein denaturation indicated a potential anti-inflammatory effect. The results revealed 51.74 ± 1.25% inhibition of Cur and 56.47 ± 1.17% of S-Cur-SMEDDS against BSA compared to diclofenac sodium (standard drug), which had 49.37 ± 1.59% inhibition. Figure 4c shows an increase in the absorbance of S-Cur-SMEDDS, indicating protein stabilization by inhibiting heat-induced denaturation. S-Cur-SMEDDS (0.25 ppm) was found to have a greater anti-inflammatory effect compared to a reference standard (diclofenac sodium) in a concentration-dependent manner. Anti-inflammatory activity using ovalbumin also showed similar results of 54.27 ± 0.69% inhibition for Cu and 62.50 ± 1.14% inhibition for S-Cur-SMEDDS compared to the standard drug (53.18 ± 1.05%). It was concluded from the above results that S-Cur-SMEDDS have good anti-inflammatory effects and could be used for the treatment of IBD [39].

### 3.9. Safety Studies of Cur SMEDDS via Acute Oral Toxicity

**Figure 5:**
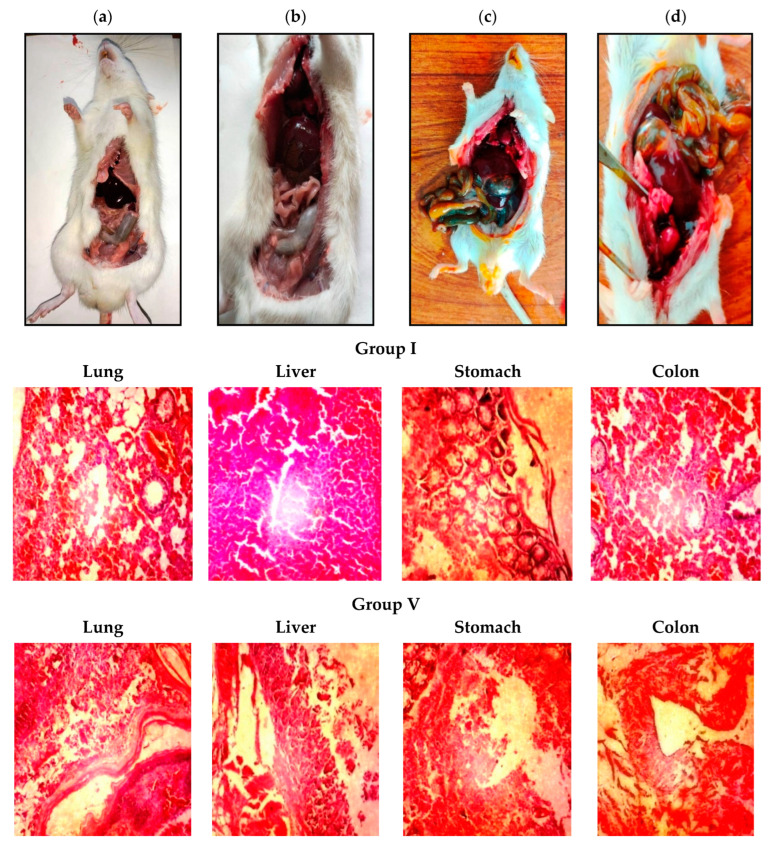
Physical examination of mice after (**a,b**) low and medium doses and (**c,d**) high dose in acute toxicity analysis. Microscopic evaluation of tissue histology of normal Group I mice organs and Group V mice treated with a lethal or toxic S-Cur-SMEDDS dose. 10× magnification.

In the acute toxicity study, after observing for 14 days, Group I and III mice were seen engaged in normal activities, such as climbing around in their cage, etc. (Figure 5). Group II mice (blank SMEDDS) treated with a dose of 8 mg/kg remained alive and normal but mice receiving a 15 mg/kg dose were dead within 24 h. In Group IV, there was a minor change in the body weights of mice with medium doses (0.2 and 0.4 g/kg) relative to the initial (*p*> 0.05) weight, but this returned to normal after 4 to 5 days of the initial dose. All animals treated with high doses (2 g/kg and 4 g/kg in mice) exhibited different behaviors from normal. In Group I, mice moved more slowly and squeezed or curled themselves against the walls or into the corners of their cages a few minutes after dosing, as shown in Figure 5. Group V mice died within 24 h of administration of a single high dose (2 g/kg and 4 g/kg) of S-Cur-SMEDDS. No significant change in body weight, tissue damage, and mortality in low-medium-dose-treated mice was observed, thus favoring the effectiveness and non-toxic nature of Cur SMEDDS at a dose of 0.4–0.8 g/kg body weight [40].

#### 3.9.1. Organs Weight Ratio and Histopathological Analysis

Organs weight ratio is an overwhelmingly effective parameter for determining in vivo toxicity. Table 2 shows that S-Cur-SMEDDS in high doses resulted in significant changes in the lungs, liver, and stomach as compared to control in acute oral toxicity evaluation. All other treatment groups do not render any specific changes. Moreover, the histopathological evaluation of different organs in Figure 5 illustrates necrosis and focal ulcerations in the stomach after treatment with a toxic dose (Group V). Furthermore, inflammation in the liver, alveolar blood cells in the lungs, congestion of vesicles in the kidneys, and parenchymal congestion in the colon were also observed in Group V. There was no significant necrosis and no severe abnormal pathology of the heart or intestine in any treated group mice [41].

#### 3.9.2. Hemolysis Assay

Hemolytic activity and potential toxicity of Cur and S-Cur-SMEDDS against human erythrocytes were examined at five different concentrations (50, 100, 200, 400, and 600 mM) after 60 min at a temperature of 37 °C (Figure 4d). The results indicated that with increasing S-Cur-SMEDDS concentration, % hemolysis also increased in a dose-dependent manner. Cur demonstrated low hemolysis (9.46 ± 1.02%) at all concentrations, even up to high concentrations. Likewise, Cur SMEDDS at a very high concentration of 600 mM showed 15.65 ± 1.32% hemolysis, which indicated the safety of Cur against RBCs after incorporation into SMEDDS, even at high concentrations. The non-toxic behavior was attributed to the selection of excipients. Tween 80 being non-ionic was considered less toxic as compared to cationic and anionic surfactants.

### 3.10. In-Vivo Macroscopic Efficacy Analysis

#### 3.10.1. Average Body Weight (ABW) Change and DAI Score

**Figure 6:**
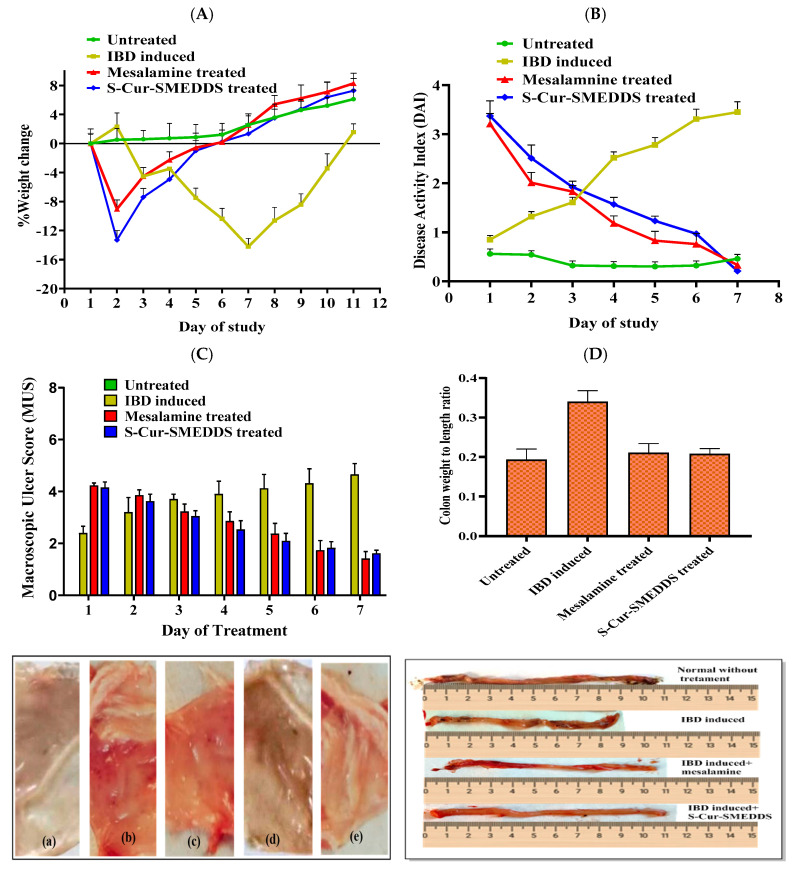
(**A**) % Body weight change in Group I (control), Group II (IBD-induced), Group III (IBD (induced + mesalamine-treated), and Group IV (IBD-induced + S-Cur-SMEDDS-treated). (**B**) Effect of S-Cur-SMEDDS on disease activity index (DAI) compared to Group II (mean ± SD) (*n* = 6). (**C**) Results of macroscopic ulcer score (MUS) after IBD induction and treatment (*p*< 0.05) in 7 days (*n* = 6) along with images of colonic mucosa: (**a**) Group I; (**b**,**c**) Group II; (**d**) Group III; and (**e**) Group IV. (**D**) Colon weight (g)-to-length (cm) ratio showing a thicker and nodular wall with shorter length in IBD-induced rat compared to Group I (*n* = 6).

The ABW of Group I (normal) kept on a normal diet and water was 191 ± 4.56 g (*n* = 6), and for Group II, the ABW after IBD induction decreased significantly (*p*< 0.05) for 7 consecutive days from 209 ± 3.42 g to 149 ± 2.85 g and then recovered slowly by day 10 (201 ± 2.87 g (*n* = 6)). For Group IV, treated with S-Cur-SMEDDS at a dose of 15 mg/kg/day, ABW was increased to 188 ± 4.53 g on day 2 after the onset of IBD (*n* = 6) and to 193 ± 2.87 g on day 4 when compared to Group I rats (Figure 6A). Group III, treated with mesalamine kept as standard, also showed comparable results. DAI was calculated for macroscopic confirmation of induction of IBD before the initiation of treatment. The DAI score of acetic acid-induced rats (Group II) was significantly higher than that of normal untreated Group I rats. From the fourth day onwards, Group II suffered from moderate anal bleeding along with loose stools (Figure 6B), whereas characteristic alterations in weight loss, stool consistency, and bleeding were gradually improved in S-Cur-SMEDDS-treated Group III and Group IV rats [42].

#### 3.10.2. Macroscopic Ulcer Score (MUS)

The effect of S-Cur-SMEDDS on MUS in rats when given immediately after the induction of IBD is shown in Figure 6C. The mucosal surface of Group I was normal, with a score 0, and Group II showed hyperemic and ulcerative signs in the colon, triggering inflammation after the induction of IBD. Scoring illustrated a significant (*p*< 0.05) increase in MUS from 0 (control) to 3.20 ± 0.56, 3.90 ± 0.49, and 4.65 ± 0.42 after 2, 4, and 7 days, respectively (*n* = 6). On the other hand, S-Cur-SMEDDS at a dose of 15 mg/kg/day (Group IV) caused a significant decrease in MUS in 7 days from 4.15 ± 0.21 to 1.62 ± 0.12 (*n* = 6) over 7 days of treatment, comparable to mesalamine at a dose of 2 mg/kg/day (Group III), which caused a decrease in MUS from 4.23 ± 0.09 to 1.42 ± 0.27 (*n* = 6) (Figure 6C).

#### 3.10.3. Cur Reversed Damage to Colon Length-to-Weight Ratio

The colon length-to-width ratio is a characteristic indicator of IBD. The total increase in ratio was reversed in the treated Groups (III, IV). Acetic acid (1% *v*/*v*) damaged the structure of the colon and induced ulcers and hemorrhagic lesions. Shortened length also resulted in the increased weight of the colon (Figure 6D). In induced Group II, the average ratio was high (0.34 ± 0.02 as compared to 0.17 ± 0.038 observed in normal Group I). An average colon-to-weight ratio (0.21 ± 0.014) was observed in Group III and Group IV, showing the protective effect on colon length and weight. Cur, as a natural drug, when loaded into SMEDDS, reversed inflammation due to increased solubility, permeation, and antioxidant effects, with minimum side effects [43].

### 3.11. Histological Confirmation According to MiSC

**Figure 7:**
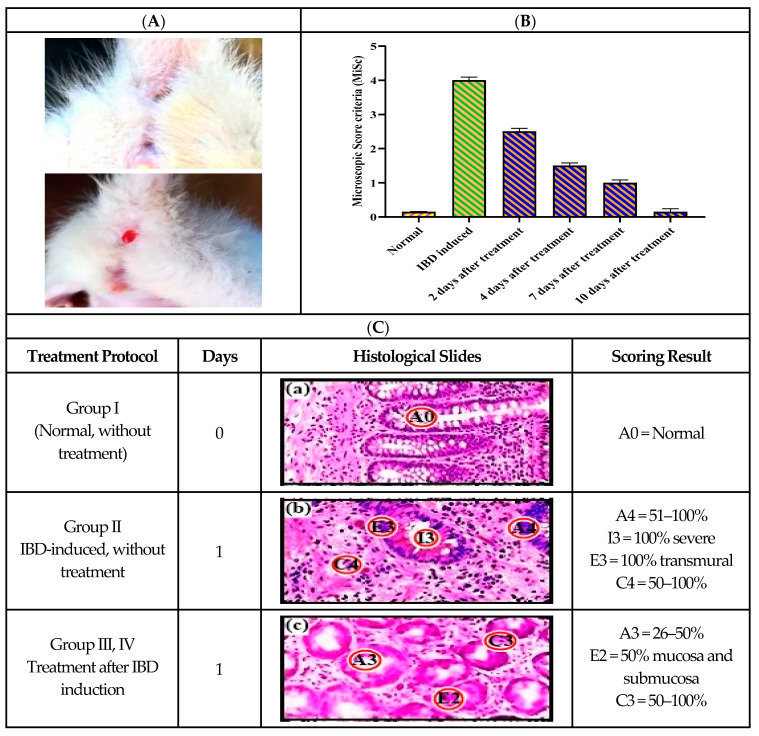

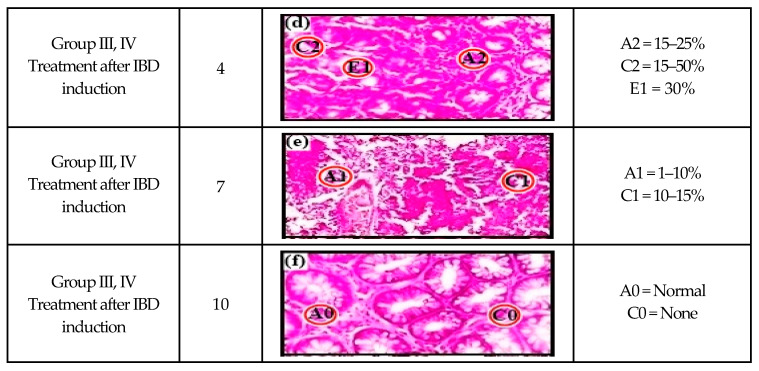
(**A**) Images of normal and anal bleeding rats after IBD induction through 1% *v*/*v* acetic acid solution. Effect of Cur-SMEDDS on the severity of inflammatory reactions in rats of Group IV for a total of 10 days. (**B**) Microscopic scoring criteria (MiSC) after 10 days of treatment (*n* = 6). (**C**) Histopathological scoring showing a description of the colon tissues of four groups on the basis of % area involved (denoted by A): inflammation, extent and Cryptosis are denoted by I, E and C. a is normal without treatment, b is IBD-induced without treatment, c, d, e and f are treatment after IBD induction.

Figure 7A shows that administration of Cur-SMEDDS reduced lymphatic infiltration in the colon of Group IV rats with acetic acid-induced IBD, comparable to Group III (mesalamine treated), Group II (IBD-induced), and Group I (normal). The results showed that MiSC was 0 in the control Group I (no IBD, without any treatment), which was increased from 0 to 3 and from 3 to 4 in the IBD-induced Group II without treatment for 7 days. Interestingly, it was reversed to an almost normal score of 0 at day 7 at an S-Cur-SMEDDS dose of 15 mg/kg/day. The possible mechanism whereby Cur confers the treatment of IBD (Table 3) is the improvement of the oxidant/antioxidant balance in colonic tissue, along with an enhanced anti-inflammatory effect after loading into SMEDDS. So, combining the lipidic properties of lipid-based nanocarriers, i.e., SEMDDS and the anti-inflammatory and antioxidant effects of Cur, increases the anti-inflammatory effect of S-Cur-SMEDDS in IBD [44].

## 4. Conclusions

Curcumin (Cur), containing a solid self-microemulsifying drug delivery system (S-Cur-SMEDDS), was successfully developed and evaluated for the treatment of inflammatory bowel disease (IBD). Physicochemical (FTIR, DSC, XRD) and morphological characterization (AFM, SEM) confirmed the stability of S-Cur-SMEDDS, while in vivo acute toxicity studies with organs weight ratio and % hemolysis (15.65 ± 1.32%) demonstrated its safety at 0.2–0.4 g/kg bodyweight Cur dose. The study was further elaborated by in vitro free radical scavenging activity (26.95 ± 1.31% to 54.06 ± 1.47%) and anti-inflammatory activity (56.47 ± 1.17%) to demonstrate the beneficial antioxidant effect of SMEDDS. Conclusively, Cur, when incorporated into SMEDDS, significantly enhances its therapeutic efficacy for the treatment of acetic acid-induced IBD in albino rats through macroscopic evaluation of colon damage aided by histopathological analysis. This suggests that S-Cur-SMEDDS could be a promising therapeutic strategy for IBD management.

## Figures and Tables

**Table 1:** Weight loss, stool consistency, and presence of stool bleeding scores for calculation of the disease activity Index (DAI) in acetic acid-induced IBD.

Score	Weight Loss (WL)	Stool Consistency (SC)	Bleeding (BL) in Stool
0	No loss or gain	Hard stool	Absent
1	1–5	Soft stool	Absent
2	5–10	Loose stool	Present
3	10–15	Mild watery diarrhea	Present
4	>15	Gross diarrhea	Gross bleeding

**Table 2:** Relative organs weights of mice in acute toxicity analysis after 14 days of Cur-SMEDDS administration (*n* = 6).

Organs	Group I Normal (g)	Group III Low Dose (g)	Group IV Medium Dose (g)	Group V High Dose (g)
Liver	2.33 ± 0.13	2.37 ± 0.51	2.42 ± 0.32	3.21 ± 0.12
Lungs	0.68 ± 0.04	0.70 ± 0.05	0.72 ± 0.01	1.14 ± 0.03
Stomach	1.76 ± 0.21	1.69 ± 0.11	1.77 ± 0.23	2.27 ± 0.20
Kidney	0.21 ± 0.02	0.24 ± 0.08	0.26 ± 0.12	1.02 ± 0.03
Heart	0.14 ± 0.01	0.15 ± 0.04	0.13 ± 0.01	0.28 ± 0.01
Intestine	8.19 ± 0.37	8.32 ± 0.56	8.23 ± 0.43	8.97 ± 0.41

**Table 3:** Histopathological scoring of IBD-based anatomic changes in colonic tissue membrane.

Anatomic Parameters	Scoring (0–4)		
Percentage of area involved (A)	A0 = Normal A1 = 1–10%	A2 = 15–25% A3 = 26–50%	A4 = 51–100%
Inflammation (I)	0 = None I1 = 30% slight	I2 = 50% moderate I3 = 100% severe	
Extent (E)	0 = None E1 = 30% mucosa	E2 = 50% mucosa and submucosa E3 = 100% transmural	
Cryptosis(C)	C0 = None C2 = 15–50%	C1 = 10–15% C3 = 50–100%	

## Data Availability

The original contributions presented in the study are included in the article/Supplementary Material. Further inquiries can be directed to the corresponding authors.

## Supplementary Materials

The following supporting information can be downloaded at: https://www.mdpi.com/article/10.3390/pharmaceutics16111406/s1, Figure S1: Relationship of average globule size with polydispersity index (PDI) of formulation S-Cur-SEDDS.
