# Correction: Ameer et al. Treatment of Inflammatory Bowel Disease by Using Curcumin-Containing Self-Microemulsifying Delivery System: Macroscopic and Microscopic Analysis. *Pharmaceutics* 2024, *16*, 1406

**DOI:** 10.3390/pharmaceutics17070810

**Published:** 2025-06-23

**Authors:** Nabeela Ameer, Muhammad Hanif, Ghulam Abbas, Muhammad Azeem, Khalid Mahmood, Dure Shahwar, Ahmed Khames, Essam Mohamed Eissa, Baher Daihom

**Affiliations:** 1 Department of Pharmaceutics, Faculty of Pharmacy, Bahauddin Zakariya University Multan, Multan 60800, Pakistan; nabeela.ameer@multanust.edu.pk (N.A.); azeem@hamdard.edu.pk (M.A.); dureshahwar77777@gmail.com (D.S.); 2 Department of Pharmacy, Multan University of Science and Technology, Multan 60800, Pakistan; 3 Department of Pharmaceutics, Faculty of Pharmaceutical Sciences, Government College University Faisalabad, Faisalabad 38000, Pakistan; ghulamabbas@gcuf.edu.pk; 4 Institute of Chemical Sciences, Bahauddin Zakariya University, Multan 60800, Pakistan; 5 Department of Pharmaceutics and Industrial Pharmacy, College of Pharmacy, Taif University, Taif 21944, Saudi Arabia; a.khamies@tu.edu.sa; 6 Department of Pharmaceutics and Industrial Pharmacy, Faculty of Pharmacy, Beni-Suef University, Beni-Suef 62514, Egypt; essam.mohamed@pharm.bsu.edu.eg; 7 Department of Pharmaceutics and Industrial Pharmacy, Cairo University, Cairo 11562, Egypt; baher.daihom@pharma.cu.edu.eg; 8 Pharmaceutical Engineering and 3D Printing (PharmE3D) Lab, Division of Molecular Pharmaceutics and Drug Delivery, College of Pharmacy, University of Texas at Austin, Austin, TX78712, USA; 9 College of Pharmacy, American University of Iraq-Baghdad, Baghdad 10071, Iraq

## Additional Affiliation

In the published publication [1], there was an error regarding the affiliations for Baher Daihom. In addition to affiliations 7 and 8, the updated affiliations should include College of Pharmacy, American University of Iraq-Baghdad, Baghdad 10071, Iraq. The authors state that the scientific conclusions are unaffected. This correction was approved by the Academic Editor. The original publication has also been updated.
