# Supplementary material for: Treatment of Inflammatory Bowel Disease by Using Curcumin-Containing Self-Microemulsifying Delivery System: Macroscopic and Microscopic Analysis
Source: Pharmaceutics. 2024 Oct 31;16(11):1406. doi: 10.3390/pharmaceutics16111406 (PMC11597465; doi:10.3390/pharmaceutics16111406)
Supplement: Supplementary file 1 [file pharmaceutics-16-01406-s001.zip › pharmaceutics-3146925-supplementary.pdf]

Supplementary material

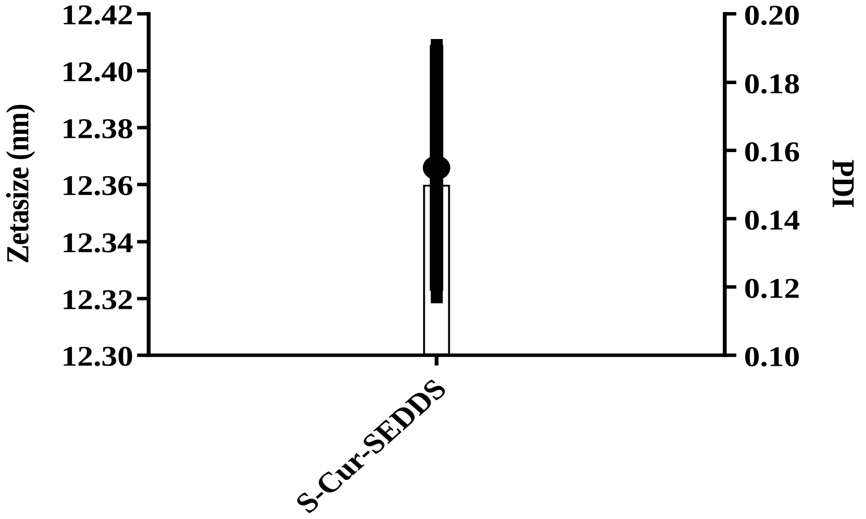

Figure S1: Relationship of average globule size with polydispersity index (PDI) of formulation S-Cur-SEDDS
